# Women's Decision-Making Power on Modern Family Planning Use and Its Associated Factors in Northwest Ethiopia

**DOI:** 10.1155/2022/9060809

**Published:** 2022-07-12

**Authors:** Yonas Deressa Guracho, Birtukan Yaregal Belay, Agaje Alemayehu, Gebremeskel Birhanie, Yared Mulu Gelaw, Mulatu Agaje, Dula Ayana, Tesfamaryam G/Meskel G/Eyesus

**Affiliations:** ^1^Bahir Dar University, College of Medicine and Health Science, Department of Psychiatry, Bahir Dar, Ethiopia; ^2^School of Computing and Information Technology, University of Wollongong, Wollongong, Australia; ^3^Pawe Health Sciences College, Pawe, Ethiopia; ^4^Bahir Dar University, College of Medicine and Health Sciences, Department of Physiotherapy, Bahir Dar, Ethiopia; ^5^Bahir Dar University, College of Medicine and Health Sciences, Department of Public Health, Bahir Dar, Ethiopia; ^6^Assosa University, College of Health Sciences, Assosa, Ethiopia; ^7^Faculty of Humanities, Bahir Dar University, Bahir Dar, Ethiopia

## Abstract

**Introduction:**

Poor decision-making power on family planning among married women is a public health concern. Despite this, there is a scarcity of research done on decision-making power of family planning use as one of their basic human rights. The study is aimed at determining the magnitude of married women's decision-making power on family planning use and its associated factors.

**Methods:**

This was a community-based cross-sectional study that was conducted on married women from May, 01-30/2021. A multistage systematic random sampling technique was applied to select 620 eligible study participants. The study used semi-interviewer questionnaires to collect data, and the collected data were entered into EpiInfo version 3.7.2 and then exported to SPSS version 20 for analysis. Bivariate and multivariable logistic regression analyses were used. The strength of associations of variables was described by using odds ratio, 95% confidence level, and *P* values less than 0.05.

**Results:**

A total of 620 women were interviewed with 98% of the response rate. Overall, married women's decision-making power on family planning was 440 (71.0%). Odds of decision-making power on family planning use were higher among women who have primary education (AOR = 11.31, CI: 4.90-26.09) and secondary and above (AOR = 6.99, CI: 3.89-12.56) as compared with those who have no education. Husbands with secondary and above educational level (AOR = 3.27, CI: 1.58-6.78), having good knowledge about family planning use (AOR = 2.41, CI: 1.48-3.95) and having a good attitude towards family planning (AOR = 6.59, CI: 4.01-10.75), had higher odds of decision-making power on family planning.

**Conclusion:**

Women's educational status, knowledge, and attitude increased the odds of decision-making power on family planning. Therefore, the authors recommend awareness creation on family planning considering lower educational level as a priority to improve women's decision-making power.

## 1. Introduction

Empowering women in decision-making is one of the sustainable development goals to be achieved by 2030 [1]. Women's decision-making power to decide freely on the number, spacing, and timing of giving birth is a basic human right [2, 3]. It is also considered as the cornerstone of reproductive health rights [4] and helps to improve quality of life of women [5].

Globally, unwanted pregnancies have been increasing. This can have serious consequences for women, families, and the communities [6]. Each year in sub-Saharan Africa, approximately 14 million unwanted pregnancies occur and the highest proportion is due to poor use of family planning [7]. On the one hand, convincing the husband about family planning use has paramount importance for a woman to achieve her contraceptive target. On the other hand, women are less empowered to overtly use contraceptives when their husbands oppose family planning [8]. The magnitude of women's decision-making power of family planning across different literature ranges from 28% to 64%, and the lowest is detected in the Ethiopian Demographic and Health Survey report of 2016 [9–15].

Even though joint decision-making with spouses about family planning has significant importance [16], women are underrepresented in decision-making power on child spacing worldwide [17]. In Ethiopia, among the top causes of perinatal mortality is pregnancy termination [18]. The most common reason women mentioned for nonuse of family planning was husband objections [15]. This makes empowering women to control their fertility has been an ongoing and daunting challenge [19].

Strengthening women's voices on family planning requires gendered collaboration and attitudinal change [20], especially, for Ethiopian women who are further disadvantaged to exercise their autonomy. However, there is very limited information regarding married women's decision-making power on family planning use and its associated factors.

## 2. Methods

### 2.1. Study Design, Study Area, Period, and Population

This community-based cross-sectional study was conducted among married women in the Metekel zone, Benishangul Gumuz Region, from May 1 to 30/2021. Metekel zone is located 570 km northwest of Addis Ababa, and it is the largest zone in the region. In Pawe town, there are 5 private clinics, one government health post, and one hospital that provide modern family planning. Women with reproductive age group (15-49 years old) in the selected villages were the study populations.

### 2.2. Eligibility Criteria

Married women who lived in Pawe town for at least six months were included. On the other hand, married women who were not able to give consent during the data collection period and those who were not living with their husbands at the time of data collection were excluded.

### 2.3. Study Variables

Dependent variable is as follows: married women decision-making power on modern FP use.

Independent variables are as follows: sociodemographic factors (age, religion, educational level, income, number of children, and occupation), husband-related factors (husband occupation, husband educational status, and husband awareness on FP use), and personal-related factors (knowledge and attitude towards family planning and household decision-making power).

### 2.4. Sample Size Determination and Sampling Method

To determine the sample size, a single population proportion formula was used with the following assumptions: the proportion of married women's decision-making power of modern family planning was 52% [11], with a 95% confidence level and 5% margin of sampling error.

(1) $$\begin{array}{c} n=\frac{{\left(Z\alpha /2\right)}^{2}\left[p\left(1-p\right)\right]}{d^{2}},\\ n=\frac{{\left(1.96\right)}^{2}\left(0.52\right)*\left(1-0.52\right)}{\left(0.05\right)2}=384\text{ then adding }10\%\text{nonresponse rate the result gives }422. \end{array}$$

Considering multistage 422 × 1.5 (design effect), the final sample was 633.

**Figure 1:**
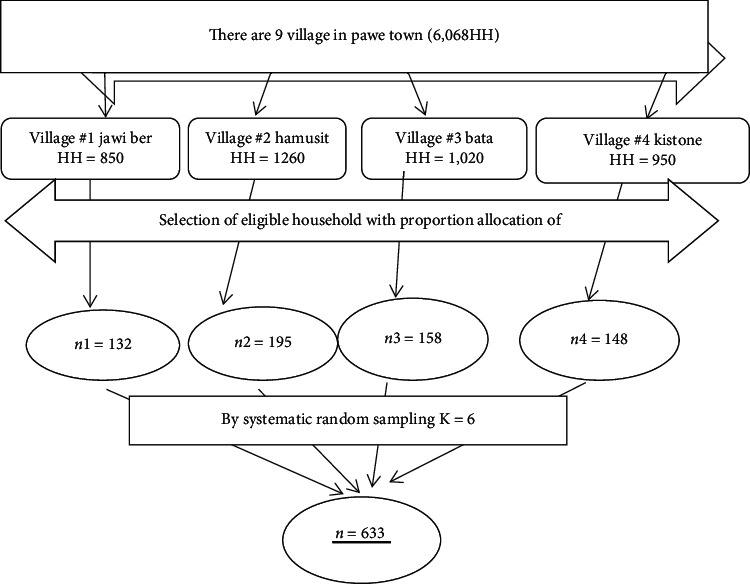
Schematic presentation of proportional allocation of sample.

Regarding the sampling method, four villages were selected randomly using lottery method. Then, individual households in the chosen kebele were selected using a systematic random sampling technique after identifying an initial starting household by use of random numbers. The sample sizes were distributed to each kebele proportional to the household size of each kebele. Eligible women in the selected household were selected and interviewed. The first household (random start) was selected by simple random sampling by taking the household list serial number between one to the respective *K* value, and the next was selected by adding the “*K*” interval of the first selected household and then subsequent households; the selection has followed similar procedures until the desired sample size was achieved (Figure 1).

### 2.5. Data Collection Procedures, Quality Control, and Analysis

Data were collected by a semistructured questionnaire. All data required were quantitative and collected by face-to-face interviews. To ensure data quality, the questionnaire was first developed in English and translated into Amharic language and translated back into English by two independent language experts and professional experts in the field of reproductive health to check the consistency. A pretest was carried out on 5% of the respondents (32 married women) in the unselected sub-Kebele (Gilgel Belese Town) two weeks before the data collection period. The data were strictly checked for completeness, accuracy, clarity, and consistency by the supervisor and principal investigator on a daily basis.

The data were first entered into EpiInfo 3.7.2 version and exported to SPSS version 20 for analysis. Data recording, categorizing, merging, computing, and counting were done before data analysis commencement. Both bivariate and multivariable logistic regression models were used to identify factors associated with women's decision-making power. Multivariable logistic adjusted odds ratios (AORs) with their corresponding 95% confidence intervals were used to assess the strength of associations between the outcome and confounding variables at *P* value < 0.05.

### 2.6. Operational Definitions

*The decision-making power of women on family planning*: it was defined in relation to women's ability to freely decide individually and decide jointly with their partners about family planning using six questions; a score less than the mean is considered as no decision-making power while scoring equal or greater than the mean score was considered as decision-making power [11]. *Household decision-making power*: on household decision-making participation, a woman, who scored below the mean on six (6) household-related questions of Ethiopian demographic health survey tools 2016, was considered as having poor participation; equal or greater than the mean was considered as good household decision-making participation.

*Knowledge on family planning*: knowledge of contraceptive methods was measured by using ten questions; all are related to contraceptive methods, and having correct answers for at least 70% was considered as good knowledge on family planning, and results less than 70% were considered poor knowledge [11].

*Attitudes on family planning*: three Likert scale items were used to measure attitude to a contraceptive method with a possible response of (agree, disagree, or neutral). A score above 70% was considered as having a good attitude, and results less than 70% will be considered as a poor attitude [21].

### 2.7. Ethical Considerations

Ethical clearance was obtained from the ethical review committee of Pawe Health Science College. Informed consent was obtained from each respondent after a detailed explanation of the study objective. The right to withdraw from the research process at any point in time was respected. Privacy and confidentiality were maintained throughout the interview.

## 3. Results

### 3.1. Characteristics of Study Participants

A total of 620 women were interviewed, and the response rate was 98%. The mean age of the study participants was 29.07 years (±6.36 S.D). The majority of respondents 392 (63.2%) were orthodox Christians. Four hundred twenty-one (67.9%) women were housewives, and 94 (15.2%) were merchants. Regarding educational status, about 43.9% (272) of women were educated up to elementary level and 25.0% (155) of the study participants had no formal education. The majority of respondents were Amhara 418 (67.4%) followed by Agew 59 (9.5%). Five hundred sixty-one (90.5%) women have children, and 445 (71.8%) women were intended to have children (Table 1).

Regarding their husbands' educational status, about 36.1% were educated up to elementary and 131 (21.1%) finished college/university. One hundred fifty-one (24.4%) were government employees followed by merchants (20.5). About 86.1% (534) of their husbands have awareness about family planning use, and 311 (50.2%) women had good knowledge about family planning use. Accordingly, about 388 (62.6%) married women have a good attitude towards family planning. More than half of the respondents are currently using family planning (53.4%), while 480 (77.4%) women are ever used to family planning. More than half of the respondents, i.e., 367 (59.2%), had autonomy on household decision-making power (Table 2).

### 3.2. Married Women's Decision-Making Power in Modern Family Planning Use

Six hundred twenty married women were interviewed for decision-making power on family planning with six-point Likert questionnaires. The reliability of the tool and the internal consistency was tested, and the Cronbach alpha was 0.781. Overall, married women's decision-making power was 440 (71.0%).

**Figure 2:**
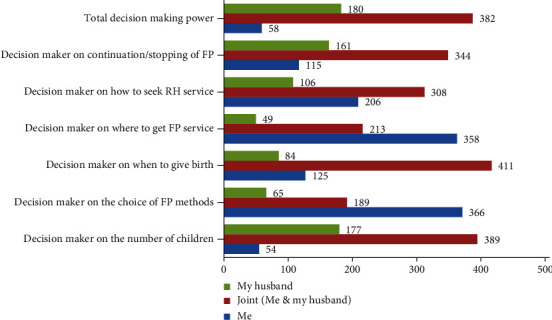
Characteristics of married women's decision-making power on the modern family planning use.

Related to decision-making power on family planning, out of 620 participants, 54 (8.71%) made decisions on the number of children by themselves; 389 (62.75%) decided jointly with their husbands. Regarding the decision-making power on the choice of family planning methods, 366 (59.03%) respondents have decided by themselves, and about 57.75% (358) of married women autonomously decided the place to attend the family planning service. Accordingly, 206 (33.226%) women have decided by themselves on reproductive health service use (Figure 2).

### 3.3. Bivariate and Multivariable Analyses

Variables that fulfill *P* value < 0.2 in the bivariate analysis underwent multivariable analysis. Multivariable analysis was performed by entering the regression variable selection method, and variables with *P* value < 0.05 were statistically significant. The goodness of model fitness was tested; the result of the Hosmer and Lemeshow test was 0.876.

Women who have secondary level and above education were about eleven times more likely to have women's decision-making power on family planning use than those women who cannot write and read (AOR = 11.31, CI: 4.90-26.09); women who have elementary level education were about seven times more likely to have women's decision-making power on family planning utilization than those respondents with no education (AOR = 6.99, CI: 3.89-12.56). On the other hand, women's husbands who have secondary level and above education were three times more likely to give a chance for women's decision-making power on family planning use than those who cannot write and read (AOR = 3.27, CI: 1.58-6.78). Regarding knowledge about family planning, those who had good knowledge were two times more likely to have decision-making power on family planning than women with poor knowledge about family planning (AOR = 2.41, CI: 1.48-3.95). Those women who had a good attitudes about family planning were seven times more likely to have decision-making power on family planning than poor attitudes about family planning (AOR = 6.59, CI: 4.01-10.75) (Table 3).

## 4. Discussion

Married women remain victims of the perpetual effects of the problem with decision-making power on family planning. This study is aimed at assessing the prevalence of married women's decision-making power on family planning use and its associated factors in Pawe Woreda, Benishangul Gumuz Region, 2021.

This study showed that the overall magnitude of married women's decision-making power on family planning use was 440 (71.0%). The result of this study is much lower than the cross-sectional study done in Indonesia (93.9%) [9] and in Basoliben Woreda Northwest Ethiopia (80%) [22]. However, it is slightly higher than the study done in Mizan-Aman, southwest Ethiopia (67.2%) [13]. The discrepancy of the results across the study might be due to the study area, i.e., the current study area, and Pawe town is semiurban which is different from the other study areas in terms of culture, norms, and ethics. On the other hand, for instance, a study conducted in Indonesia has a wider difference in the perception of norms and ethics, specificity of family planning, study population, and study setting.

Women empowerment in decision-making power appears to have a modest impact on modern family planning use [23]. In this study, about 10% of married women had their decision-making power on modern family planning 58 (9.4%). The current result was lower compared to another study conducted in Northwest Ethiopia (14.2) [22], EDHS 2016, where 22% of married women made their decision mainly by themselves. The result of this study is higher than the study done in Nigeria where only 6.2% of the women reported making their own decisions. Lack of discussion with the partner on family planning use was a predictor that women are subordinate and dependent on their husbands; this hinders women's rights not to confidently exercise her human and democratic rights.

Strengthening women's voices requires gendered collaboration and attitude change; education is among the best tools to empower women in multidisciplinary activities to tackle poverty [20]. This study showed women who had secondary level and above education were about eleven times more likely to have decision-making power on family planning use than those who cannot write and read; women who had elementary level education were about seven times more likely to have decision-making power on family planning use than those respondents. This article is in line with other articles such as a study conducted at Mizan-Aman, Ethiopia [13], in Nigeria [24, 25].

Regarding husband educational level, the current study showed that husbands who had secondary level and above education were three times more likely to empower their wives for their decision-making power on family planning use than those who cannot write and read. This is congruent with other studies in Mizan-Aman, Ethiopia [13], in sub-Saharan Africa countries, namely, Burkina Faso, Mali, Niger, and Chad [26]. Equal decision-making is a predictor and shown to negotiate a democratic right and human rights and resulted from the positive impact of educational policy, particularly in empowering women.

Regarding basic knowledge about FP use, those who had good knowledge were two times more likely to have decision-making power on family planning use than those with poor knowledge. The finding of the study is in line with the study conducted in Basoliben, Ethiopia [22], and Gedeo zone Ethiopia [21]. Women are less empowered to decide on family planning if they have poor knowledge about it, and by virtue, the probability of convincing their husbands is less likely. Therefore, empowering women to know about family planning is of paramount importance to raise the level and consistent contraceptive use.

Those women who had a good attitudes about FP use were seven times more likely to have decision-making power on FP use than those with poor attitudes about FP use (Table 2). Other cross-sectional studies done in Ethiopia also support this finding [15, 21]. Women's attitude is a key element in the use of family planning and has also a temporal relation with the intention to use contraceptives besides having decision-making power on family planning. This is therefore the poorer attitude of women to family planning, the lesser to decide either alone or jointly with their husbands.

## 5. Conclusion

Married women's decision-making power on modern FP use was low compared to the desired right of women. Educational level of women and their husbands, good knowledge about family planning use, and good attitude towards family planning use were factors associated with the married women's decision-making power on modern FP use.

## 6. Limitation

This study is only confined to married women; however, future researchers would contribute a lot if they involve men who are in union and justify the associated factors with a qualitative study design.

## Figures and Tables

**Table 1:** Sociodemographic characteristics of married women's decision-making power in modern family planning use and its associated factors in Pawe town, northwest Ethiopia, 2021 (*n* = 620).

Variables	Count	Women decision-making power	
		No	Yes
Age of women			
15–24	157 (23.3)	28 (15.6)	129 (29.3)
25–29	176 (28.4)	31 (5.0)	145 (23.4)
30–34	127 (20.5)	47 (7.6)	80 (12.9)
≥35	160 (25.8)	74 (11.9)	86 (13.9)
Educational level of women			
Cannot write & read	155 (25.0)	116 (64.4)	39 (8.9)
Elementary	272 (43.9)	51 (28.3)	221 (50.2)
Secondary	114 (18.4)	10 (5.6)	104 (23.6)
College/university	79 (12.7)	3 (1.7)	76 (17.3)
Religion			
Orthodox	392 (63.2)	101 (16.3)	291 (46.9)
Muslim	120 (19.4)	48 (7.1)	72 (11.6)
Protestant	87 (14.0)	24 (3.9)	63 (10.2)
Catholic	21 (3.4)	7 (1.1)	14 (2.3)
Ethnicity			
Amhara	418 (67.4)	125 (20.2)	293 (47.3)
Agew	59 (9.5)	13 (2.1)	46 (7.4)
Kambata	51 (8.2)	19 (3.1)	32 (5.1)
Hadiya	42 (6.8)	13 (2.1)	29 (4.7)
Oromo	28 (4.5)	6 (1.0)	22 (3.5)
Other^∗^	22 (3.5)	4 (0.6)	18 (2.9)
Women's occupation			
Housewife	421 (67.9)	153 (24.7)	268 (43.2)
Merchant	94 (15.2)	18 (2.9)	76 (12.3)
Govt employee	86 (13.9)	3 (0.5)	83 (13.4)
Daily labor	19 (3.1)	6(1.0)	13 (2.1)
Total	620	180 (29)	440 (71)
Having children			
No	59 (9.5)	15 (2.4)	44 (7.4)
Yes	561 (90.5)	165 (26.6)	396 (63.9)

Other^∗^: Shinasha, Gumuz, and Tigre.

**Table 2:** Coupled and personal-related characteristics of married women's decision-making power on modern family planning use and its associated factors, in Pawe town, northwest Ethiopia, 2021 (*n* = 620).

Variables	Count	Women decision-making power	
		No	Yes
Educational level of husband			
Cannot write & read	110 (17.7)	77 (12.4)	33 (5.3)
Elementary	224 (36.1)	72 (11.6)	152 (24.5)
Secondary	155 (25.0)	21 (3.4)	134 (21.6)
College/university	131 (21.1)	10 (1.6)	121 (19.5)
Husband occupations			
Govt employee	151 (24.4)	12 (1.9)	139 (22.4)
Merchant	127 (20.5)	39 (6.3)	88 (14.2)
Private employee	112 (19.7)	35 (5.6)	87 (14.0)
Farmer	99 (16.0)	42 (6.8)	57 (9.2)
No job	10 (1.6)	1 (0.1)	9 (1.5)
Husband family planning awareness			
No	86 (13.9)	75 (12.1)	11 (1.8)
Yes	534 (86.1)	105 (16.9)	429 (69.2)
Current family planning use			
No	289 (46.6)	118 (19.0)	171 (27.6)
Yes	331 (53.4)	62 (10.0)	269 (43.4)
Ever family planning use			
No	140 (22.6)	85 (13.7)	55 (8.9)
Yes	480 (77.4)	95 (15.3)	385 (62.1)
Intention to have child			
No	175 (28.2)	71 (11.5)	104 (16.8)
Yes	445 (71.8)	109 (17.6)	336 (54.2)
Household decision-making			
No	253 (40.8)	116 (26.8)	87 (14.0)
Yes	367 (59.2)	14 (2.3)	353 (56.9)
Knowledge about family planning			
Poor knowledge	309 (49.8)	133 (21.5)	176 (28.4)
Good knowledge	311 (50.2)	47 (7.6)	264 (42.6)
Attitude towards family planning			
Poor attitude	232 (37.4)	137 (22.1)	95 (15.3)
Good attitude	388 (62.6)	43 (6.9)	345 (55.6)

**Table 3:** Bivariate and multivariable analyses of associated factors of married women's decision-making power in modern family planning utilization in Pawe town, northwest Ethiopia, 2021.

Variables	Women decision-making power			AOR (95% CI)	*P* value
	No (*n* = 180)	Yes (*n* = 440)	COR (95% CI)		
Age of women					
15–24	28	129	1	1	0.217
25–29	31	145	1.02 (0.58-1.78)	1.98 (0.93-4.26)	
30–34	74	80	0.37 (0.21-0.64)	1.26 (0.59-2.67)	
≥35	47	86	0.25 (0.15-0.42)	1.01 (0.50-2.04)	
Women's educational level					
Cannot write & read	116	39	1	1	
Elementary	51	221	12.89 (8.08-20.69)	6.99 (3.89-12.56)^∗^	<0.001
Secondary & above	13	180	41.18 (21.08-80.45)	11.31 (4.90-26.09)^∗^	
Husband educational level					
Cannot write & read	77	33	1	1	
Elementary	72	152	4.63 (3.01-8.01)	1.56 (0.82-2.98)	0.004
Secondary & above	31	255	19.19 (11.05-33.35)	3.27 (1.58-6.78)^∗^	
Intention to have child					
Yes	109	336	1	1	0.71
No	71	104	2.10 (1.45-3.05)	0.89 (0.51-1.59)	
Knowledge about family planning use					
Good knowledge	54	283	1	1	
Poor knowledge	126	157	4.25 (2.89-6.23)	2.41 (1.48-3.95)^∗^	<0.001
Attitude towards family planning					
Good attitude	43	345	1	1	
Poor attitude	137	95	11.57 (7.67-17.45)	6.59 (4.01-10.75)^∗^	<0.001

^∗^Significantly associated variables at multivariable analysis.

## Data Availability

Data is available from corresponding author upon the request.
